# A win ratio approach to comparing ordinal or non-normal outcomes in clinical trials

**DOI:** 10.1186/1745-6215-14-S1-O47

**Published:** 2013-11-29

**Authors:** Duolao Wang, Stuart Pocock

**Affiliations:** 1 London School of Hygiene and Tropical Medicine, London, UK

## 

Clinical trials are often designed to compare an ordinal outcome such as New York Heart Association (NYHA) functional classification or a non-Normal outcome such as duration of hospital stay in cardiovascular trials. The conventional statistical method for such a comparison is a non-parametric Mann-Whitney test, which provides a *P*-value for testing the hypothesis that the distributions of both treatment groups are identical but does not provide an estimate of treatment effect. For that, Hodges and Lehmann proposed estimating the shift parameter between two populations and its confidence interval. However, such a shift parameter does not have a straightforward interpretation and its confidence interval contains zero in some cases when Mann-Whitney test produces significant result.

To overcome the above problems, we introduce the concept of the win ratio for analysing such data. The original use of the win ratio was for a hierarchy of composite time to event outcomes (Pocock et al 2012). Patients in the new and control treatment are formed into all possible pairs. For each pair the new treatment patient is labelled a "winner" or a "loser" if it is known who had a favourable outcome. The win ratio is the total number of winners divided by the total numbers of losers. A 95% CI and *P*-value for the win ratio are readily obtained. This method is demonstrated by analyses of NYHA in PARTNER B trial and hospital stay in PLACIDE trial. Statistical properties of the win ratio statistic are investigated using simulation studies of non-Normal outcomes under various scenarios.
